# Lingual Bronchogenic Cyst: A Case Report and Literature Review of a Rare Pathology

**DOI:** 10.7759/cureus.78451

**Published:** 2025-02-03

**Authors:** Boyu Ma, Qingcong Zeng, John Le, James Wicker, Kathlyn Powell

**Affiliations:** 1 Oral and Maxillofacial Surgery, University of Alabama at Birmingham, Birmingham, USA; 2 Oral and Maxillofacial Surgery, University of Florida College of Medicine-Jacksonville, Jacksonville, USA; 3 Anatomic Pathology, University of Alabama at Birmingham, Birmingham, USA

**Keywords:** oral cavity tumors and cyst, oral medicine, oral pathology, pediatric oral and maxillofacial surgery, tongue lesions

## Abstract

Choristomas are rare, benign embryonic tumors characterized by normal tissue appearing in abnormal sites. When lined with respiratory epithelium, they are classified as bronchogenic cysts. While these cysts most commonly occur in the mediastinum or other thoracic regions, their occurrence on the tongue is exceedingly rare. Here, we present the case of a four-year-old boy diagnosed with a bronchogenic cyst of the tongue, highlighting its clinical and histological features, diagnosis, and management, along with a review of the literature.

## Introduction

Bronchogenic cysts are benign congenital lesions with normal tissue occurring in abnormal sites. They are part of a group of tumors called choristomas, heterotopic cysts, or foregut duplication cysts [1]. Choristomas can be histologically characterized based on their derivative tissue. Bronchogenic cysts are derived from the abnormal budding or branching of epithelial cells during the development of the tracheobronchial tree or associated vasculature. The majority of cases (>99%) occur in the mediastinum and lung, while extrathoracic sites are exceedingly rare [2,3]. Here, we describe a case of a lingual bronchogenic cyst arising in the ventral tongue of a four-year-old male. The cyst was completely excised, and no recurrence was seen at follow-up.

## Case presentation

A healthy four-year-old male, with no prior medical history, presented with a two-week history of an enlarging tongue nodule on the ventral surface of his tongue. The nodule initially presented at birth without symptoms, but became enlarged and painful. On examination, a well-circumscribed, firm nodule was palpated at the midline ventral tongue with tenderness. The overlying mucosa had a purple tinge, but no other abnormalities were noted intraorally. The remaining oral and head and neck examination was without any remarks. No imaging modalities were utilized. Based on the clinical examination, the differential diagnosis included a branchial cyst, thyroglossal duct cyst, dermoid cyst, lipoma, and ranula. A total excisional biopsy of the mass was performed one week later under general anesthesia.

Intraoperative findings showed a cystic mass that was successfully dissected circumferentially and removed in its entirety from the underlying tongue musculature. Primary closure was achieved following the removal of the mass. The patient had an uneventful recovery and was discharged home on the same day as the surgery.

**Figure 1:**
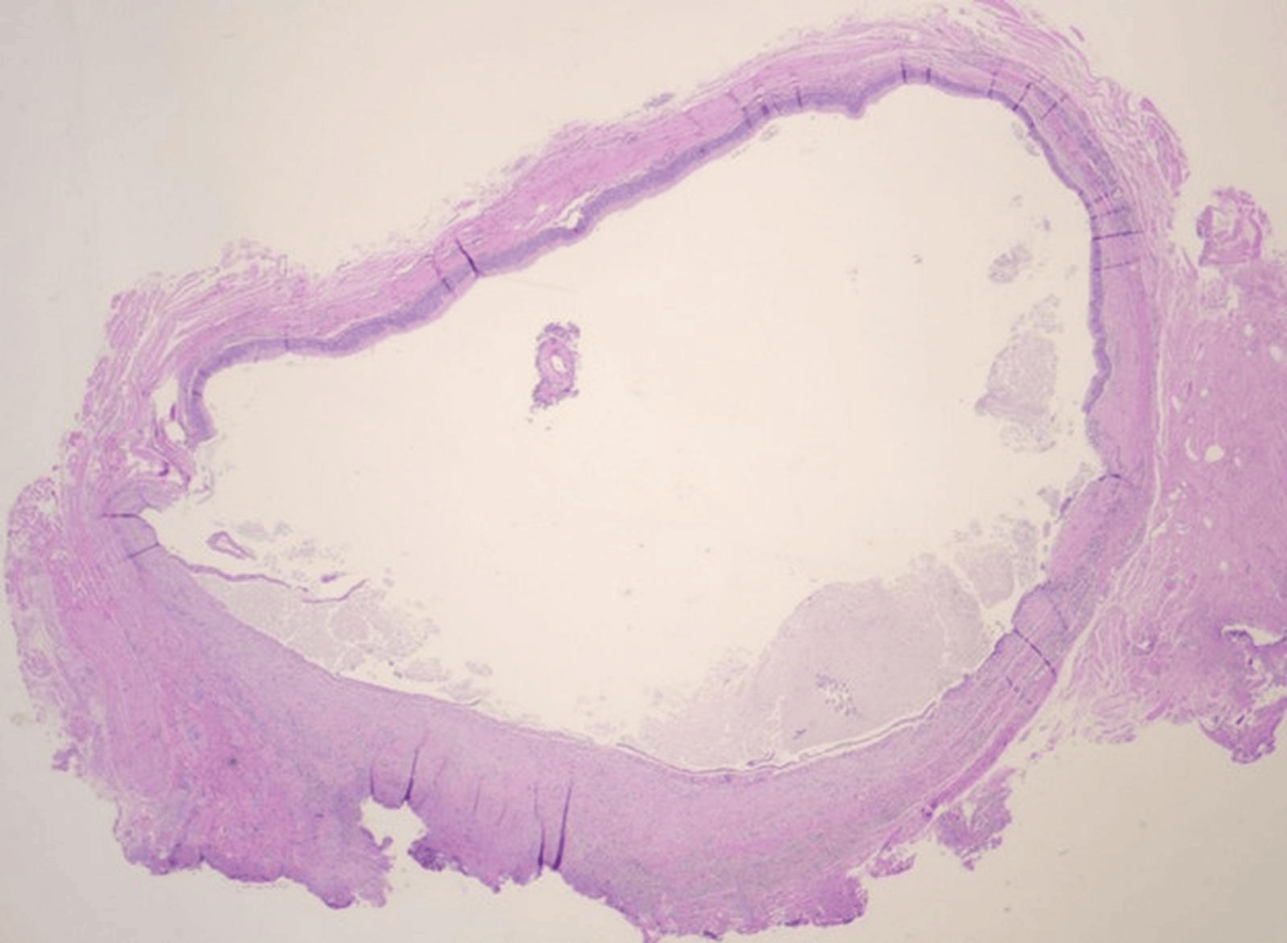
Low-power (20x) histopathology of the bronchogenic cyst showing a cystic structure with patchy mixed inflammation. (Hematoxylin and Eosin stain)

**Figure 2:**
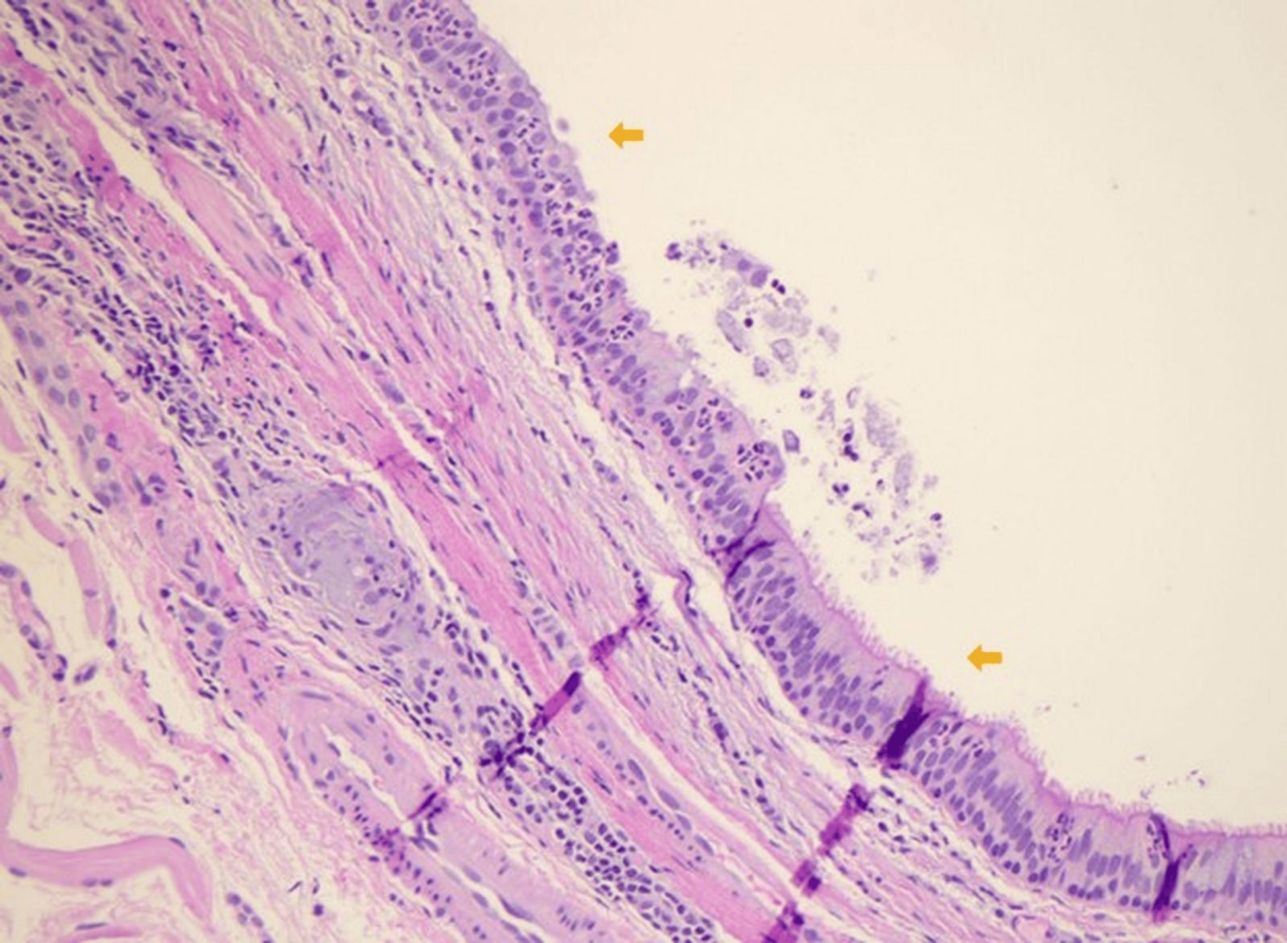
High-power (40x) histopathology of the bronchogenic cyst wall. The arrows indicate a pseudostratified ciliated columnar epithelium (hematoxylin and eosin stain).

**Figure 3:**
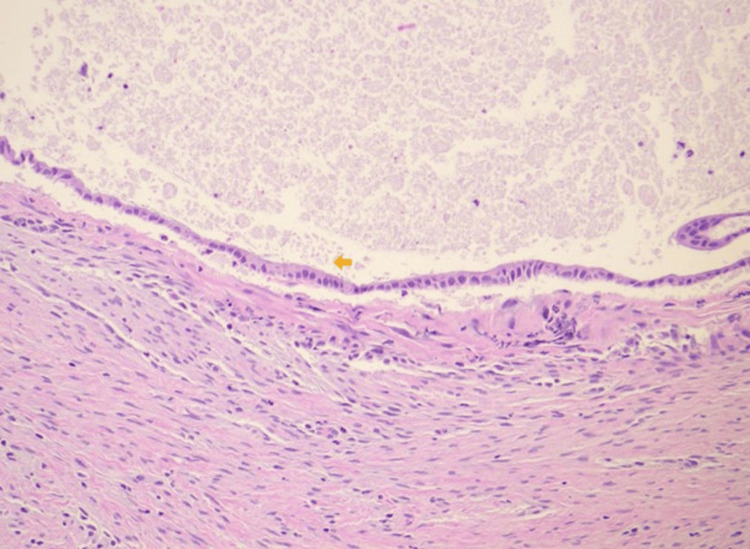
High-power (40x) histopathology of the bronchogenic cyst wall. The arrow indicates foci of the low cuboidal epithelium (hematoxylin and eosin stain).

**Figure 4:**
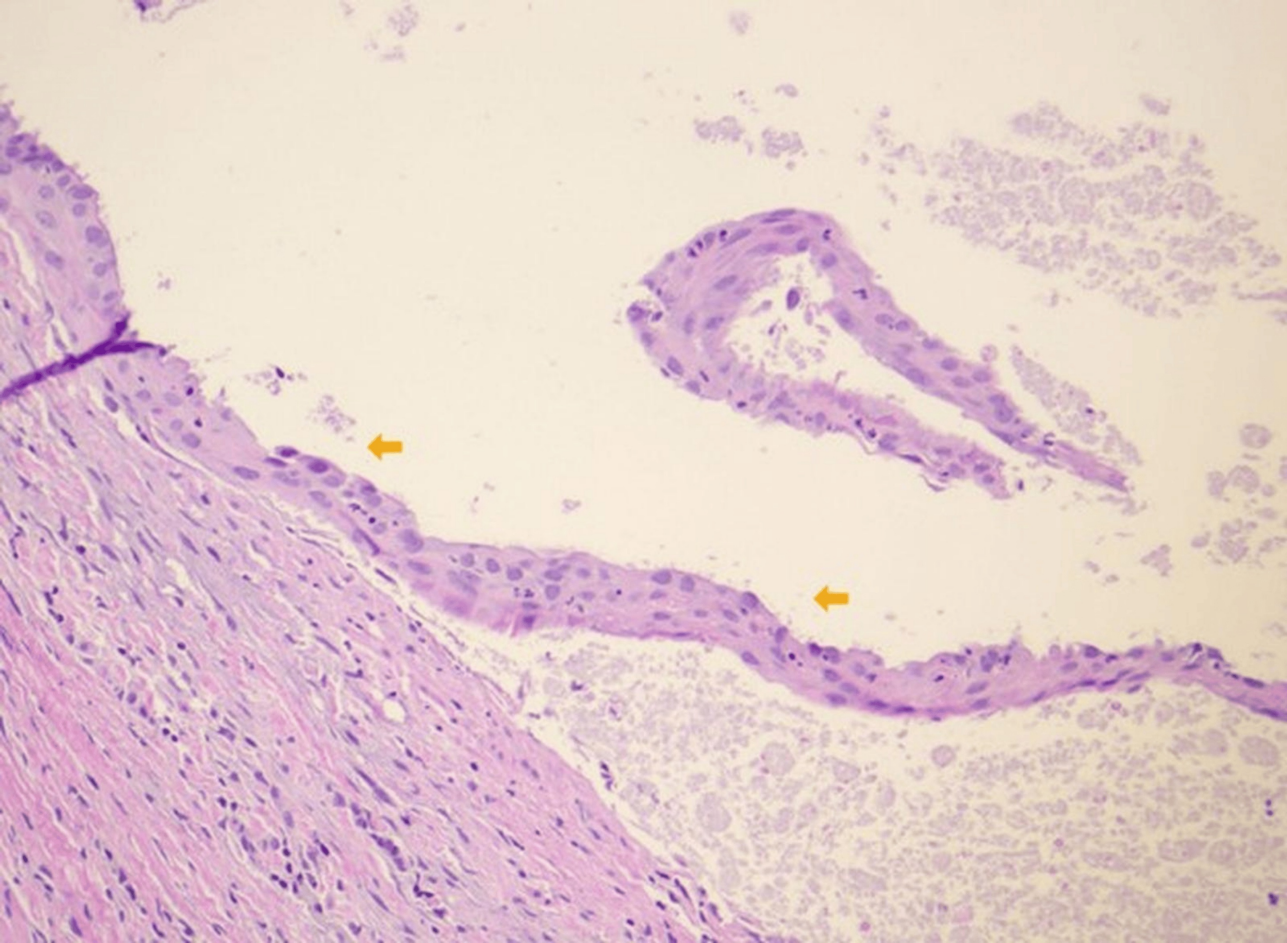
High-power (40x) histopathology of the bronchogenic cyst wall. The arrows indicate foci of squamous metaplasia (hematoxylin and eosin stain).

The gross specimen was gray-pink, 0.7 cm x 0.6 cm x 0.6 cm in size, and contained a tan-brown purulent fluid. Histological examination showed a cystic lesion predominantly lined by pseudostratified ciliated columnar epithelium with patchy mixed inflammation noted (Figures 1, 2). There were foci of low cuboidal epithelium and squamous metaplasia, consistent with bronchogenic cysts (Figures 3, 4) [2]. A pathologic diagnosis of a bronchogenic cyst was made.

The patient returned for an examination two weeks postoperatively. The ventral tongue was well healed, and the patient reported no sensorineural or functional deficits. Since the recurrence rate for bronchogenic cysts is low and the oral tongue can easily be surveilled, a one-year follow-up appointment was scheduled for the patient.

## Discussion

The exact etiology of bronchogenic cyst development remains unknown. During the third week of embryogenesis, the foregut differentiates into the respiratory tract ventrally and the gastrointestinal tract dorsally [1-4]. The tongue develops during the fourth week, forming from the pharyngeal endoderm, branchial mesoderm, and occipital somites. As a result, the primitive foregut and developing branch arches are in close proximity. Bronchogenic cysts can develop from undifferentiated remnants from the primitive tongue that are entrapped during tongue development [4-10]. The presentation of bronchogenic cysts in early life suggests a developmental aberrancy as the underlying cause. One hypothesis proposes that the cysts form from small buds of diverticula that separate from the foregut during tracheobronchial tree development [3].

**Figure 5:**
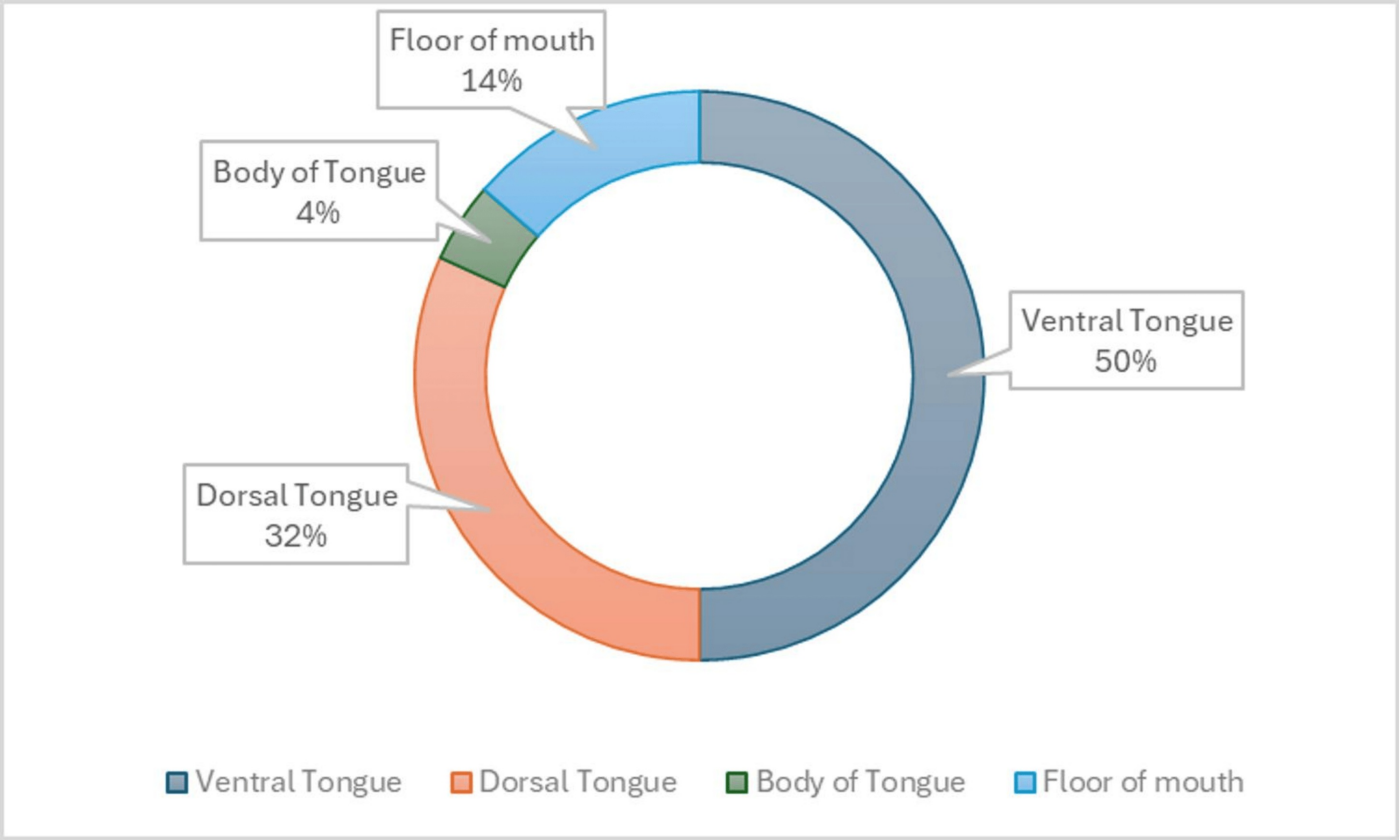
Locations of bronchogenic cysts reported intraorally in the literature. Generated from intraoral sites from Table 1 with Microsoft Excel.

The most common sites for bronchogenic cyst development are in the mediastinum and lung (75% and 25%, respectively). The incidence of this cyst in the head and neck region, however, is less than 1% of cases [5-9]. Even rarer is a lingual bronchogenic cyst, with less than 30 cases being reported. Most of the cases demonstrate a male predilection with an onset age of six years or younger. While these are present at birth, they can present as late as 61 years of age (Table 1). The most common location is the ventral tongue (Figure 5). Lingual bronchogenic cysts are typically noticed at birth clinically presenting as asymptomatic lingual masses or with feeding difficulties during infancy. If sufficiently large, they may be detected during prenatal ultrasonography screening [3]. In our case, the bronchogenic cyst presented initially as an enlarging mass that eventually became indurated and painful.

**Table 1:** Review of case reports of lingual bronchogenic cysts from 1963 to 2023.

Author	Age	Sex	Site	Clinical history	Surgery
Gunnartodir et al. (2018) [1]	4 years old	Male	Ventral tongue	Asymptomatic	Excision
Bailey (1982) [2]	1 day old	Male	Ventral tongue	Present since birth, difficulty in eating	Excision
Aldawood et al. (2021) [3]	6 years old	Male	Ventral tongue	Asymptomatic	Excision
Joshi et al. (2013) [4]	6 years old	Male	Dorsal tongue	Asymptomatic	Excision
Kim et al. (1998) [5]	27 years old	Male	Dorsal tongue	Asymptomatic	Excision
Volchok et al. (2007) [6]	61 year old	Male	Ventral tongue	Transformation to adenocarcinoma	Excision
Fink (1963) [7]	5 years old	Male	Dorsal tongue	Painless swelling, difficulty in eating	Excision
Constatinides et al., (1982) [8]	9-month-old female	Female	Ventral tongue	Present since birth, difficulty in eating	Excision
Boue et al. (1994) [9]	4 years old	Male	Ventral tongue	Asymptomatic	Excision
Manor et al. (1999) [10]	11 years old	Male	Body of tongue	Macroglossia, difficulty in eating	Excision
Azanero et al. (2009) [11]	4 years old, 21 years, old	Male	Ventral tongue, Dorsal tongue	Present since birth, difficulty in breastfeeding, difficulty in eating	Excision
Boffano et al. (2009) [12]	35 years old	Female	Floor of mouth	Asymptomatic	Excision
Soares et al. (2011) [13]	12 years old	Female	Ventral tongue	Present since 6 years old, slow growth, difficulty in eating	Excision
Chai et al. (2011) [14]	6 months old	Female	Ventral tongue	Difficulty in eating	Excision
Juneja et al. (2011) [15]	1 year old	Female	Dorsal tongue	Present since birth, difficulty in eating	Excision
Fortier et al. (2013) [16]	17 years old	Female	Dorsal tongue	Asymptomatic	Excision
Kwak et al. (2014) [17]	2 years old	Female	Ventral tongue	Asymptomatic	Excision
Kün-Darbois et al. (2015) [18]	9 days old, 12 months old	Male and female	Dorsal tongue	Asymptomatic	Excision
Peters et al. (2018) [19]	10 years old, 27 years old	Male and female	Floor of mouth	Asymptomatic	Excision
Cialente et al. (2020) [20]	44 years old	Male	Floor of mouth	Present since 9 months old, swelling on the body of the tongue	Excision
Current case (2023)	4 years old	Male	Ventral tongue	Present since birth	Excision

Multiple authors have reported the use of magnetic resonance imaging (MRI) and computed tomography (CT) to characterize bronchogenic cysts and define the boundaries for surgical excision [1,3]. While these imaging modalities aid in characterizing the mass, they do not provide a definitive diagnosis. The differential diagnosis for a bronchogenic cyst includes other developmental anomalies, such as branchial cysts, thyroglossal duct cysts, and other foregut duplication cysts [2]. Additionally, more common head and neck pathologies, such as lipomas, mucoceles, ranulas, vascular malformations, dermoid cysts, and teratomas, must also be considered [3-8].

On histopathological analysis, all reported cases of bronchogenic cysts have been shown to contain pseudostratified columnar epithelium [4-12]. Focal areas of non-ciliated cuboidal, columnar or stratified squamous epithelium [13-18]. The presence of cartilage and smooth muscle is often seen [3]. Additionally, mild chronic inflammation has been reported in the majority of cases, as presented in this case [3-8].

With regard to treatment, the majority of the cases reported were managed with surgical excision alone (Table 1). Surgical excision is considered the definitive treatment [12-17]. Furthermore, there have been no documented cases of the role of adjuvant therapy following surgical excision [16-20]. In general, there is no recurrence following complete excision of the cysts in both short- and long-term follow-up periods [10-20]. However, there have been cases of adenocarcinoma, arising from intra-thoracic bronchogenic cysts [6]. Volchok et al. described a case of adenocarcinoma arising from a previously undiagnosed lingual foregut duplication cyst in a 61-year-old man [6]. In this case, the adenocarcinoma was treated and monitored according to the standardized cancer guidelines.

## Conclusions

Lingual bronchogenic cysts are extremely rare foregut choristomas that are typically diagnosed within the first decade of life. In the head and neck region, the majority generally present asymptomatically on the ventral tongue. These cysts are managed with surgical excision with a low recurrence rate. The defining histopathologic characteristic distinguishing this cyst from other common cysts in the head in neck is a pseudostratified ciliated columnar epithelium, which is consistent with respiratory tissue. As with many conditions in the oral cavity, early recognition of the disease process is important in treatment.
